# Explaining variance of avian malaria infection in the wild: the importance of host density, habitat, individual life-history and oxidative stress

**DOI:** 10.1186/1472-6785-13-15

**Published:** 2013-04-08

**Authors:** Caroline Isaksson, Irem Sepil, Vladimer Baramidze, Ben C Sheldon

**Affiliations:** 1 Department of Zoology, Edward Grey Institute, University of Oxford, Oxford, United Kingdom; 2 Current address: Evolutionary ecology group, Department of Biology, Lund University, Lund, Sweden

**Keywords:** Antioxidants, Glutathione, Host density, Hydroperoxides, Oxidative stress, *Parus major*, *Plasmodium*

## Abstract

**Background:**

Avian malaria (*Plasmodium sp.*) is globally widespread, but considerable variation exists in infection (presence/absence) patterns at small spatial scales. This variation can be driven by variation in ecology, demography, and phenotypic characters, in particular those that influence the host’s resistance. Generation of reactive oxygen species (ROS) is one of the host’s initial immune responses to combat parasitic invasion. However, long-term ROS exposure can harm the host and the redox response therefore needs to be adjusted according to infection stage and host phenotype. Here we use experimental and correlational approaches to assess the relative importance of host density, habitat composition, individual level variation and redox physiology for *Plasmodium* infection in a wild population of great tits, *Parus major*.

**Results:**

We found that 36% of the great tit population was infected with *Plasmodium* (22% *P. relictum* and 15% *P. circumflexum* prevalence) and that patterns of infection were *Plasmodium* species-specific. First, the infection of *P. circumflexum* was significantly higher in areas with experimental increased host density*,* whereas variation in *P. relictum* infection was mainly attributed to age, sex and reproduction. Second, great tit antioxidant responses – total and oxidizied glutathione - showed age- , sex- and *Plasmodium* species-specific patterns between infected and uninfected individuals, but reactive oxygen metabolites (ROM) showed only a weak explanatory power for patterns of *P. relictum* infection. Instead ROM significantly increased with *Plasmodium* parasitaemia.

**Conclusions:**

These results identify some key factors that influence *Plasmodium* infection in wild birds, and provide a potential explanation for the underlying physiological basis of recently documented negative effects of chronic avian malaria on survival and reproductive success.

## Background

Avian malaria (*Plasmodium sp*) is a globally widespread disease but considerable variation exists in both prevalence and parasitaemia at small spatial scales [1,2]. This variation can be generated by several ecological, demographical and immunological mechanisms, including variation in vector and host densities and host susceptibility. For example, temperature, altitude and proximity to water bodies are small-scale ecological factors known to influence vector (e.g., mosquitoes *Culex, Aedes,* and *Culiseta*, and black flies *Simuliidae*) and/or parasite distribution and abundance [2-8]. This in turn increases the probability of transmission from vector to host. Furthermore, if an area with malaria transmission is of poor quality (e.g., has low availability and/or quality of food), this may reduce the bird’s condition and ability to resist a parasitic infection [9-11], further compounding the rate of infection. Similarly, although transmission rate of vector borne diseases are predicted to be independent of host density [12], high densities of birds may reduce the availability of food or other resources, thereby further impairing resistance. Other demographic parameters that may influence infection rates include sex and age structures of the population arising from differences in behaviour, reproductive effort and immune capacities [13,14].

The impact of avian malaria on wild birds has been difficult to estimate, especially in areas where avian malaria is common and the host-parasites have co-evolved. However, recent long-term and experimental studies have shown that there are effects on both survival and reproductive success [15-18]. On naïve hosts such as island species and captive birds in zoo the impacts have been devastating [19]. For example on the Hawaiian Islands there was an accidental introduction of *Plasmodium relictum* and one of its vectors which caused direct die-offs during the acute infection state which continues to play a significant role in species distribution and is a serious threat for endangered species [19-22].

In the early stages of infection, the first line of defense is the host’s innate, non-specific immune defence. Immune cells generate and release reactive oxygen species (ROS, commonly referred to as oxidative burst) that attack the parasite [23,24]. The advantage of oxidative burst is that it can act within minutes after transmission, in contrast to the acquired immune defence, which needs longer to develop the target-specific antibodies. However, the non-specificity and general cytotoxicity of ROS make it impossible for the host to distinguish between self and parasite tissue, causing oxidative damage to all types of adjacent molecules, which if not repaired, can lead to haemolysis and cellular dysfunction (i.e., a surplus of oxidants to antioxidants is commonly referred to as oxidative stress). If the parasite resists the host’s ROS attack and starts to multiply and grow, it also starts to generate ROS as a by-product of haemoglobin digestion [25]. Consequently, the detrimental and beneficial effects of ROS need to be fine-tuned by the host’s antioxidant defences during different stages of infection [26,27], which can result in a quadratic relationship between oxidative stress and malaria severity. This association can depend on other host- and environment-specific characters, including age and nutrition which influences the oxidative status [28-30].

Here we assess the relative importance of ecological (habitat quality and spring date), demographic (host density, age and sex), life-history (reproduction and body mass) and physiological (total and oxidized glutathione which are the most important intra-cellular antioxidant system and reactive oxygen metabolites [ROM]) factors for natural variation in *Plasmodium* infection and parasitaemia (i.e. parasite density) in a population of great tits, *Parus major.* The sampling took place in the spring, which suggests that most infections are relapse infections from previous autumn, since most vectors have not yet emerged [31]. Assessment of the relative importance of a broad range of factors, from ecology to physiology, to explain variation in patterns of avian malaria infection in the wild has rarely been done [11], but is important for understanding the significance of effects found under controlled laboratory situations. In addition, the present population allows test of experimentally increased host density on patterns of prevalence.

## Results

In the present population of great tits, 107 of the 299 screened birds were infected with *Plasmodium* (36%); 65 with *P. relictum* (prevalence of 22%) and 44 with *P. ciricumflexum* (prevalence of 15%) morphospecies; only two birds had mixed infection (see Table 1 for sample sizes). The infection for first year breeders was 17% for both morphospecies, and the corresponding figures for older birds were 26% and 14%, for*, P.relictum* and *P. ciricumflexum,* respectively (see below for analysis of age-dependence).

**Table 1:** **Sample sizes of *Plasmodium *infected and uninfected individuals**

	***P. circumflexum***		***P. relictum***	
	**Uninfected**	**Infected**	**Uninfected**	**Infected**
Young	n = 106	n = 21	n = 105	n = 22
Older	n = 144	n = 23	n = 124	n = 43
Female	n = 140	n = 23	n = 121	n = 42
Male	n = 112	n = 21	n = 110	n = 23

### Infection of *Plasmodium relictum*

**Figure 1:**
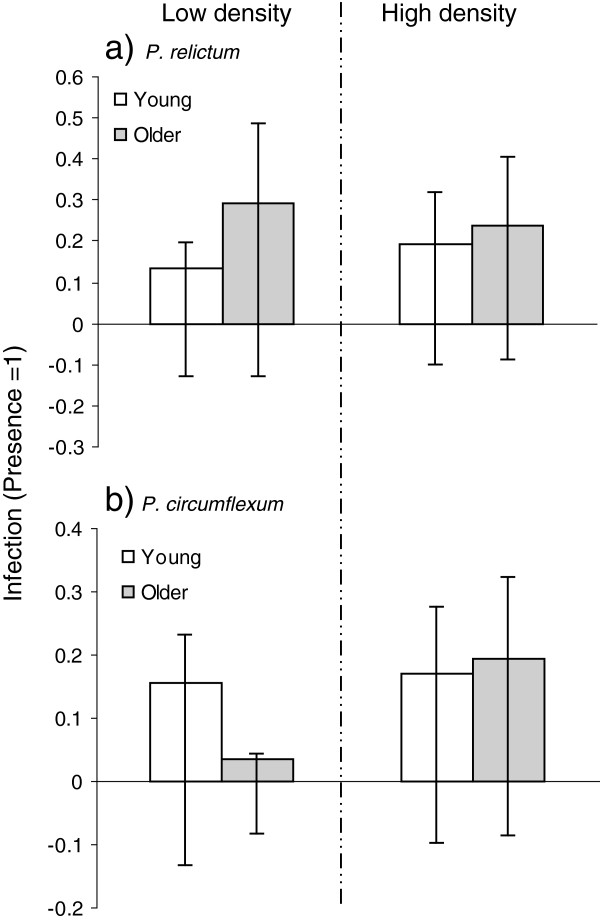
***Plasmodium *infection in relation to breeding density and age.** Infection of ***a****) P. relictum,* and ***b****) P. circumflexum* in adult great tits (first year breeders and older) in relation to experimental manipulation of breeding density (LD = low density, HD = high density). Mean ± Confidence Intervals (CI), presence = 1 and absence = 0, i.e., the higher value the larger proportion is infected.

In Table 2, the top ten models are presented along with the averaged model estimates for the parameters included (all models with Δ < 4). For *P. relictum* infection, there were only small differences in the AICc between the top ten models (Table 2), so no model could be confidently singled out as the best. However, five out of the ten parameters were important for explaining variation in *P. relictum* infection; namely age, sex, clutch size, spring date and ROM. The experimental manipulation of density was not important in any of the top ten models for *P. relictum* (Figure 1). The raw data from the top three parameters revealed a 12% increase in infection from first year to older breeders and males were less infected (17%) than females (26%). In terms of clutch size, uninfected birds had laid a larger clutch size (mean ± standard deviation: 9.23 ± 1.49) compared to the infected birds (8.88 ± 1.55). Regarding spring date, it should be mentioned that it was highly correlated to GSH:GSSG ratio ( r = - 0.293, p < 0.0001, see Additional file 1) i.e., the earlier in the spring the more reduced to oxidized glutathione.

**Table 2:** **Summary of AIC modelling of *Plasmodium relictum *infection in great tits**

	Parameters*	**Deviance**	**AICc**	**ΔAICc**	***ω***	
Model 1	1+2+9	267.23	277.47	0	0.01	
Model 2	1+2+4+8+9	263.20	277.66	0.19	0.01	
Model 3	1+2+8+9	265.40	277.74	0.27	0.01	
Model 4	1+2+4+9	265.40	277.74	0.27	0.01	
Model 5	1+2+4+8	265.61	277.95	0.48	0.01	
Model 6	2+4+8	268.04	278.28	0.81	0.01	
Model 7	2+4+8+9	266.06	278.4	0.93	0.01	
Model 8	1+2+8	268.17	278.41	0.94	0.01	
Model 9	1+2+4	268.25	278.49	1.02	0.01	
Model 10	1+2+5+9	266.17	278.51	1.04	0.01	
Null model	Intercept	276.01	280.06	2.59	0.00	
* see code below for which parameters that are included						
Averaged model parameters:						
Parameters	Code	Coeff.	SE	Low CI	Upp CI	Rel. import.
(Intercept)		2.49	3.14	-3.66	8.65	
Age(young)	1	-0.40	0.39	-1.16	0.35	0.69
Cs	2	-0.13	0.13	-0.37	0.12	0.68
D(low)	3	0.00	0.12	-0.23	0.23	0.12
Spring date	4	-0.04	0.05	-0.13	0.06	0.52
GSSG	5	0.04	0.11	-0.17	0.25	0.25
Mass	6	-0.04	0.12	-0.28	0.19	0.26
Q(low)	7	0.00	0.15	-0.29	0.29	0.13
ROM	8	-0.13	0.18	-0.49	0.23	0.54
Sex(male)	9	-0.28	0.35	-0.97	0.40	0.56
tGSH	10	0.04	0.10	-0.17	0.24	0.26

The top ten models are presented along with relative parameter importance (based on a model average with Δ AICc > 4).

*cs* clutch size, *D* breeding density, *GSSG* oxidized glutathione, *Q* habitat quality, *ROM* reactive oxygen metabolites, *tGSH* total glutathione, *Rel. imp* relative parameters importance, *CI* confidence interval (lower and upper), *ω* model weight.

**Figure 2:**
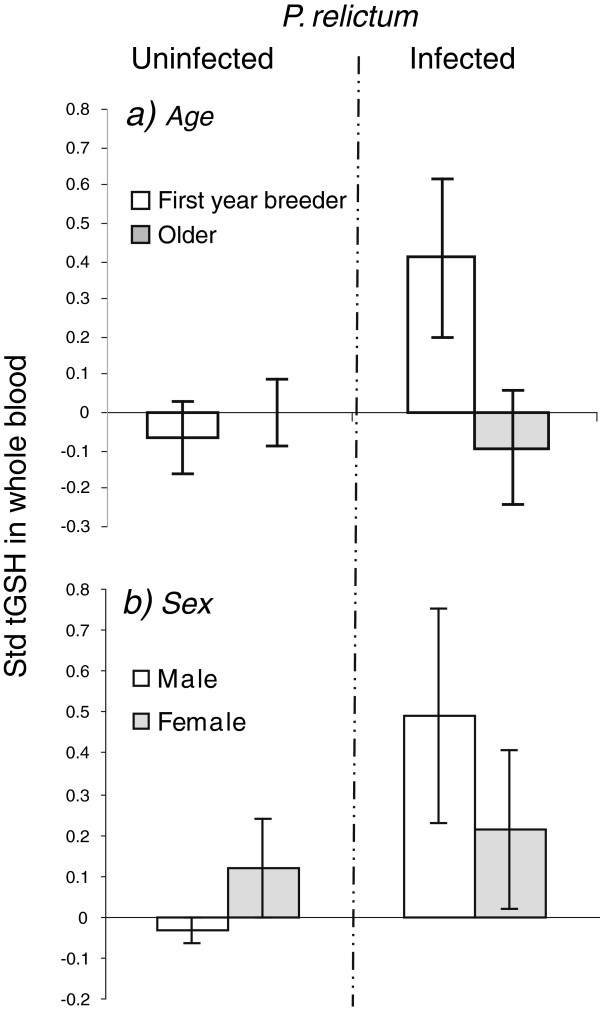
**tGSH and *P.relictum *infection.** Age- (**a**) and sex-specific (**b**) pattern in cellular glutathione (tGSH) in great tits uninfected or infected by *P.relictum.* Mean standardized tGSH ± SE are presented.

When interactions were included the top model retained tGSH, age×tGSH and sex×tGSH apart from the parameters in Model 1 (sex, age and clutch size; see S2 for model details, Figure 2). Although not significant, the interactions suggest that young and male great tits have higher tGSH when infected compared to when uninfected; and vice versa for older and female birds.

### Infection of *Plasmodium circumflexum*

**Figure 3:**
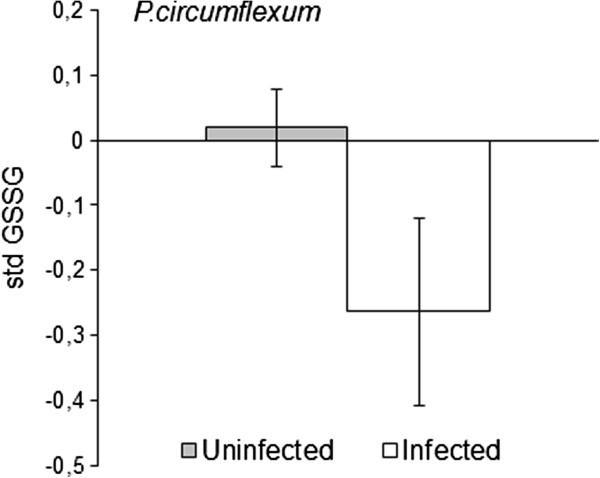
**GSSG and *P. circumflexum *infection.** Oxidized glutathione (GSSG) in *P. circumflexum* infected and uninfected great tits. Mean standardized GSSG ± SE are presented.

In Table 3, the top ten models are presented along with the averaged model estimates for the parameters included. For *P. circumflexum*, the manipulation of bird density was the best explanatory variable, followed by GSSG (Table 3). In line with our prediction, great tits breeding in high density areas showed a twofold increase in prevalence (18%) compared to birds breeding in the low density areas (9%). Moreover, *P. circumflexum* infected birds had a lower GSSG than uninfected birds (mean std GSSG ± standard deviation, uninfected: 0.01 ± 0.96, infected: -0.27 ± 0.91, Figure 3). When interactions were added, the best fitting model included density, GSSG, age and age×density (see S3 for model details, Figure 1). Closer examination revealed that the density pattern was found to be stronger in older great tits, with 3% *P.circumflexum* infection in low density compared to 19% in the high density habitats. For first year breeders the *P. circumflexum* infection was 18% in both densities.

**Table 3:** **Summary of AIC modelling of *Plasmodium circumflexum *infection in great tits**

	Parameters*	**Deviance**	**AICc**	**Δ AICc**	***ω***	
Model 1	3+5	207.98	216.14	0.00	0.07	
Model 2	3	210.49	216.58	0.44	0.05	
Model 3	1+3+5	207.27	217.52	1.38	0.03	
Model 4	3+5+8	207.47	217.72	1.58	0.03	
Model 5	3+5+10	207.57	217.82	1.68	0.03	
Model 6	1+3	209.71	217.88	1.74	0.03	
Model 7	3+4+5	207.73	217.98	1.84	0.03	
Model 8	2+3+5	207.76	218.01	1.87	0.03	
Model 9	3+8	209.98	218.14	2.00	0.02	
Model 10	3+5+9	207.90	218.14	2.01	0.02	
Null model	Intercept	226.63	230.67	4.34	0.01	
* see code below for which parameters that are included						
Averaged model parameters:						
Parameters	Code	Coeff.	SE	Low CI	Upp CI	Rel. import.
(Intercept)		-1.48	1.76	-4.93	1.97	
age(young)	1	0.07	0.21	-0.35	0.49	0.23
Cs	2	-0.01	0.05	-0.11	0.09	0.16
D(low)	3	-1.03	0.44	-1.90	-0.16	1.00
Spring date	4	0.00	0.02	-0.04	0.05	0.17
GSSG	5	-0.18	0.22	-0.61	0.24	0.59
Mass	6	-0.01	0.08	-0.16	0.15	0.14
Q(low)	7	0.01	0.16	-0.30	0.32	0.13
ROM	8	-0.02	0.08	-0.18	0.14	0.21
sex(male)	9	0.01	0.13	-0.25	0.28	0.13
tGSH	10	-0.02	0.09	-0.19	0.15	0.18

The top ten models are presented along with relative parameter importance (based on a model average with Δ AICc > 4).

*cs* clutch size, *D* breeding density, *GSSG* oxidized glutathione, *Q* habitat quality, *ROM* reactive oxygen metabolites, *tGSH* total glutathione, *Rel. imp* relative parameters importance, *CI* confidence interval (lower and upper), *ω* model weight.

In addition, parasite density of both *Plasmodium* species was quantified. However, the species-specific models did not explain any significant variation in parasitaemia, so will not be presented. There were no significant associations between species-specific parasitaemia and any of the physiological variables (F = 0.14 - 2.48, p = 0.71 - 0.11). Although we are aware of the difference in virulence of the two different *Plasmodium* species [23], the digestion of haemoglobin by both species releases iron (Fe^2+^ and Fe^3+^) which can generate ROM [24-26] (see also Methods). A correlational analysis were performed and it revealed the predicted positive association between ROM and parasitaemia (n = 93, r = 0.25, p = 0.018), but not for tGSH (n = 100, r = -0.00, p = 0.990), GSSG (n = 100, r = -0.05, p = 0.605) and the GSSG:tGSH ratio (n = 100, r = -0.14, p = 0.178).

## Discussion

In the present study, we combined experimental manipulation of host density across two contrasting environments with assessment of host physiology and life-history in a natural population of great tits to understand causes of variation in *Plasmodium* infection. The key and novel findings of this study were, firstly, that manipulation of host density increases *P. circumflexum* infection, but not infection with *P. relictum*; and secondly; that host antioxidants and ROM physiology show species-specific patterns in relation to infection, and thirdly, that ROM increases with *Plasmodium* parasitaemia.

### Avian malaria, ecology and demography

Host density was found to be the only ecological and demographic parameter of importance for *P. circumflexum* infection. This link was absent for infection with *P.relictum*. This suggests that population density reduces the bird’s ability to resist infection by *P. circumflexum*, the more virulent of the two *Plasmodium* species [32]. Possibly, this is via more intense competition over resources or it could be mediated via higher levels of testosterone or stress hormones in high density areas which have been shown to supress the immune system [33]. Alternatively, the vectors transmitting *P. circumflexum* either aggregate where hosts are abundant or that vectors carrying the parasite are able to infect several birds, whereas the vector transmitting *P.relictum* may be more widely distributed or only feed on one host at one point in time [33-35] but see [36].

In Wytham Woods; a woodland in the proximity of Bagley, recent work on great tits and the closely related sympatric host species, blue tits (*Cyanistes caeruleus*), have shown that the two *Plasmodium* species have pronounced differences in their spatial distributions and impacts on both hosts species [7,32]. While *P. circumflexum* infections exhibit pronounced spatial structuring that is stable over years in both host species; *P. relictum* infections are effectively randomly distributed in space (Lachish et al. in press). Likewise, *P. circumflexum* infections are linked with reduced survival, particularly during the acute stage of infection [32]; whereas *P. relictum* infections are associated with reproductive costs [7]. Hence the species-specific relationships between ecological factors and parasite prevalence detected in the present study are not unexpected. Moreover, in Wytham woods it was shown that territory size was linked to host density i.e., the larger territory the lower density. This estimate of host density did not explain patterns of *Plasmodium* infection in the conspecific blue tits, *Cyanistes caeruleus*[1]. Similar to the present study, however, a positive association was found between large-scale breeding density (i.e., measured as number of nests within a 1500 m radius) and *Leucocytozoons* prevalence and parasitaemia in nestling eagle owls (*Bubo bubo*) [37]. In temperate regions *Plasmodium* species show a bimodal peak in infection, one in autumn and one in spring, and the spring peak (measured here) is most often a relapse from the autumn infection gained in the previous year [31,38]. Since juvenile great tits disperse during late summer-early autumn, the impact of the current environment on first-year breeders may be small compared to the older great tits which have strong site fidelity. Indeed, the association of host density manipulation was stronger among the older great tits for infection with *P. circumflexum*. In contrast to our prediction, habitat quality and spring date did not explain much (or any) variation in infection patterns in either of the two morphospecies.

### Avian malaria, life-history and oxidative stress

There was no overall important life-history or oxidative stress parameter that explained variation in infection of both morphospecies. For *P. relictum* infection, age was one of the best predictors and in accordance to our prediction; older great tits were to a greater extent infected compared to younger great tits. However, there was no sign of an age effect on *P. circumflexum* infection, which is possibly due to the strong influence of host density (see above)*.* Naturally, the probability of getting bitten by an infected vector increases with time regardless of species [1]. Despite that, older individuals may have an acquired immune defence that is familiar to *Plasmodium* parasites, so an overall decline in cellular mechanisms and resistance with age can be an explanation for the influence of age [39]. Indeed, since most birds are likely to have a relapse infection, the low tGSH in old *P. relictum* infected birds can be a result of a more rapid decline of the antioxidant defences (or indirectly via degradation of other cellular mechanisms affecting GSH synthesis) compared to uninfected ageing birds. For first year breeders, tGSH was higher when infected compared to those that were not infected, which is what we predicted during a chronic malaria infection. Alternatively, young and old individuals have different hormetic responses to parasites [40] or selective disappearance of the old individuals with a high tGSH in response to infection. The cause for this age difference in tGSH in response to infection warrants further investigation.

Given the difference between the sexes in behaviour and physiology during the breeding season, it was not surprising that sex explained some variation in *P.relictum* infection [41]. Generally, in both mammals and birds, males are to a greater extent infected by malaria, but in the present population females have slightly higher presence of infection, but not significantly. Similarly, in a nearby forest, Wytham woods, female blue tits showed a significantly higher presence of infection of *P. relictum*[7]. Possibly, the direction the association is a result of the timing of sampling i.e., breeding season (discussed below). Furthermore, there was an interaction between sex and tGSH, revealing that infected males have a higher tGSH compared to uninfected males, whereas infected females have lower (or no difference) tGSH compared to uninfected females. Interestingly, sex shows a similar pattern to age with regards to the interaction (see above, and Figure 2). The group with relatively lower presence of infection (males and young) had higher tGSH when infected compared to the uninfected birds for the same group, whereas the group with relatively higher presence of infection (females and older) had lower tGSH when infected. This sex- and age-specific response of tGSH to *P. relictum* parasites may play a role in susceptibility to this infection.

Alternatively, and independently of the similar age pattern, the investment into reproduction by infected females is associated with a greater physiological costs compared to uninfected females and compared to males, thus, females may not be able to up-regulate their antioxidant system [11]. Recently, a study of great tits revealed that increasing brood size results in an increase in malaria parasitaemia in males, but that both sexes show a decrease in resistance to oxidative stress i.e., red blood cell resistance to an external free radical attack [42]. However, a direct link between oxidative stress and parasitaemia was not found. Here clutch size was an important predictor of *P. relictum* infection, with uninfected birds having larger clutch size than infected birds, supporting the previously found fitness cost [32]. ROM was slightly higher in the uninfected birds, thus the production of hydroperoxyl radicals when exposed to oxidizing agent is less likely in *P. relictum* prevalent birds. This seem to be in contrast to overall parasitaemia, where ROM increases with parasite density (p = 0.018), when analysed separately per species only a trend was found (*P. relitum*: p = 0.12, *P. circumflexum*: p = 0.11). As mentioned above, the overall effect of parasitaemia may be independent of parasite species virulence but rather a result of parasite metabolism [43]. When a parasite digests haemoglobin it releases haem with iron in its ferrous state (Fe^2+^). This state is highly reactive and can easily oxidize to Fe^3+^ and thereby generate ROM [43-45]. Thus, increased parasite metabolism and abundance may be followed by increased oxidative damage (due to increased generation of hydroperoxyl radicals) unless the antioxidant defence is adequate. In a recent study of Seychelles warbler (*Acrocephalus sechellensis*), malaria infection was linked to a higher ROM, but parasitaemia was not measured [11]. An alternative explanation for the patterns in both Seychelles warblers and great tits is that high generation of hydroperoxyl radicals may be linked to other unmeasured abiotic or biotic factors that increase the susceptibility to generate them, resulting in a positive association between avian malaria and ROM.

In contrast to *P. relictum*, GSSG (oxidized GSH) rather than the tGSH explained variation in *P. circumflexum* prevalence. Generally, a high GSSG indicates that the cell experiences an oxidative challenge, and that the GSH antioxidant system is in action [46]. However, in an infected cell the antioxidant efficiency of GSH has been shown to be suppressed by a down-regulation of glutathione peroxidase and a low GSSG can be detected even though the oxidative challenge is high [47], see also [48]. This is perhaps the most likely interpretation of the lower GSSG in *P. circumflexum* prevalent birds. However, it should be noted that in the present study all erythrocytes were measured i.e. not only the infected ones. Thus, the redox environment for the parasite may be different to the average host cell GSH/GSSG redox homeostasis. Possibly, the individuals with low GSSG are more susceptible to *P. cirumflexum* infection or there has been selective disappearance of individuals with high intracellular GSSG independent of infection. In captivity, healthy partridges GSSG show a quadratic relationship with age [49]. Unfortunately, the present study is not able to disentangle the effect of age and infection on GSSG and GSH. The species difference can be a result of different manipulations of the host GSH system, parasite metabolism or virulence [14,50,51].

## Conclusion

The present study is the first experimental test showing that bird breeding density increases infection of an avian malaria species - *P. circumflexum*. Patterns of *P. relictum* infection*,* on the other hand, were better explained by host factors: sex, age and clutch size. As in human malaria, GSH redox system seems to form a part of the physiological response to avian malaria, but in a species-specific pattern and depending on the bird’s age and sex. However, independent of *Plasmodium* species and host factors, *Plasmodium* abundance showed a positive association with generation of ROM. This could form part of a mechanistic explanation for the recently documented negative impact of infection on fitness in passerines [15,16,32] and suggest that oxidative stress may be an important cost of infection in wild populations. Future experimental studies will be important for determining the causal relationship between the different avian malaria species and parameters of oxidative stress.

## Methods

### Ethical consideration

All work was carried out within UK standard requirements (Home office license number PL30/2659) and the guidelines of the University of Oxford. All methods were approved by the University of Oxford ethical review board and permission to work in Bagley Woods was obtained from the property owner’s - St. John’s College/Oxford. Birds were caught by nest box traps and then kept in individual cloth bags and sampled within half an hour. All blood samples were taken by C.I (Personal license PIL 30/8503).

### Study site

This study was carried out during April-June in 2009 in a nest-box population of great tits (*Parus major*). The study site, Bagley woods, is a 250-ha woodland (51°42^′^N, 5°37^′^W), consisting of a mixed matrix of plantations of either deciduous trees (mainly oak *Quercus* dominated*,* high quality habitat = HQ, n_plots_ = 8) or evergreen trees (mainly pine *Pinus* and larch *Larix*, low quality = LQ, n_plots_ = 4) (see map in Additional file 2). High quality and low quality classification is based on abundance of caterpillars in deciduous areas, crucial food source for great tits during spring time [52]. In early 2007, nest boxes (n = 513) were experimentally placed at two densities (high density = HD, 68 nest boxes, n_plots_ = 6, and low density = LD, 18 nest boxes, n_plots_ = 6) in similar sized areas (between 9–12 ha). These arrangements produce average densities of 23.3 great tit nests per HD area and 12.3 great tit nests per LD area (i.e., an approximate two-fold increase in density). Birds nest according to the box availability, which creates plots of different bird densities. Although some natural nests are likely to occur within the study site, these are unlikely to affect the treatment densities to any great extent (only one natural nest was found, CI personal observation). In this way, a two-factor design is created, with replicated manipulations. There were no difference between the high and low breeding densities in clutch size (F = 0.89, p = 0.344), condition (F = 2.33, p = 0.128) and spring date (F = 1.06, p = 0.306). Breeding close to standing water bodies can be an important determinant for avian malaria infection due to higher abundance of vectors [1]. The only water basins within the present wood arise from sporadic truck tracks after heavy rain; these are, as far as we are aware, distributed at random throughout the wood.

### Study species and sampling

Adult great tits were captured with nest box traps while feeding their chicks (between day 6–10 after hatching). In total 299 birds were captured (n_females_= 164, n_males_ = 135, see Tab. 1). Among the older birds, 131 of 168 (78%) were known (based on capture history) to have bred in the study site the previous year (2008), and only 10 (7.6%) individuals had moved to a different density plot between 2008 and 2009. Regarding the first year breeders, 56 out of 126 (44%) had been ringed as nestlings in Bagley in the previous year. For five birds age was not determined.

Approximately 110 μl blood was drawn from the jugular vein with a heparinised syringe. For malaria screening, a few drops of blood were stored in SET buffer (0.015 M NaCl, 0.05 M Tris, 0.001 M EDTA, pH 8.0) and saved in -20°C. Then total genomic DNA was extracted using standard ammonium acetate method and stored in AE Buffer (Qiagen) until disease identification (see below). For the oxidative stress assays, two different approaches were used: for GSH assays, 40 μl blood was diluted with 2 μl EDTA and then immediately frozen in liquid nitrogen in the field. The rest (approximately 60 μl) was kept cool on ice until it was centrifuged two to three hours later, and then frozen in liquid nitrogen. For proper separation between plasma and red blood cells samples were centrifuged at 1800 rpm for 10 minutes. At the end of each field day all samples were transferred to a -80°C freezer. All the biochemical assays were run within 6 months after collection and within the same week to reduce variation in degradation among the samples [53].

### Malaria screening

A quantitative polymerase chain reaction (qPCR)-based assay was used to amplify and quantify a portion of the *Plasmodium* cytochrome b gene [15]. Total DNA concentration was measured using a Picogreen assay (Quant-iT Picogreen dsDNA Assay Kit, Invitrogen) and molecular conversion calculations, based on the size and base composition of the DNA fragment, were used to estimate DNA copy number in this solution see [40]. The samples were diluted to 2 ng/μl. qPCR amplifications were performed in 25 μl reactions, with the following final concentrations: 12.5 μl of Super Mix (Platinum SYBR Green qPCR SuperMix-UDG, Invitrogen), 10 ng of diluted DNA and 0.2 μM of primers L9 (5’-AAACAATTCCTAACAAAACAGC-3’) and New R (5’-ACATCCAATCCATAATAAAGCA-3’). The primers were carefully designed to ensure *Plasmodium*-specific amplification and to enable the differentiation of the two *Plasmodium* morphospecies that are common in the study site, *P. relictum* and *P. circumflexum* for more details see [7,32]. The temperature profile included a pre-incubation step at 50°C for 2 min and an initial denaturation step at 95°C for 2 min, followed by 43 cycles of denaturation at 95°C for 15 sec, annealing at 56°C for 30 sec and extension at 72°C for 30 sec. The reactions were run on an Mx3000P machine (Stratagene) and SYBR Green detection system was employed. Each sample was run in triplicate and *Plasmodium* copy number was estimated by calculating the mean value for the triplicate.

### Oxidative stress assays

#### Total and oxidized glutathione (tGSH and GSSG)

Glutathione (GSH) is the most abundant intra-cellular antioxidant, and probably the most important. It targets hydrogen peroxide (H_2_O_2_) which is the major oxidant produced during oxidative burst and parasite metabolism [54,55]. The oxidized form of GSH – glutathione disulphide (GSSG) can be reduced back to its active form (GSH) by a nicotinamide adenine dinucleotide phosphate (NADPH) dependent reaction and glutathione reductase (GR), and it is this redox-cycle that the quantification method of tGSH and GSSG is based on [56]. With minor modifications, tGSH and GSSG assays were conducted according to Baker et al. [56] and Vandeputte et al. [57], adapted for birds in Isaksson et al. [58]. Briefly, 4 μL of whole blood was diluted with 16 μL 5% 5-sulfoasalicylic acid (SSA). To precipitate proteins, all samples were then centrifuged at 8000 g for 10 min at 4°C. 10 μL of the sample was further diluted with 400 μL GSH buffer (143 mM NaH_2_PO_4_ and 6.3 mM EDTA, pH 7.4). For GSSG assay, 200 μl of the sample were treated immediately with 1 μl of 4-vinylpyridine (4-vp) and incubated for 1 hour at 37°C. The reaction mixture (prepared fresh daily) contain GSH buffer, 5.5’-dithio-bis (2-nitrobenzoic acid) (DTNB) and NADPH. A 96-well plate was prepared for both tGSH (diluted plasma without 4-vp) and GSSG (diluted sample incubated with 4-vp) by adding, 20 μl sample and 200 μl reaction mix, and then 2 μl of 0.34 U/ml and 0.17 U/ml GR added for tGSH and GSSG, respectively. All samples were run in duplicates and the colour generated by 2-nitro-5-thiobenzoic acid (the product from the reaction between DTNB and GSH) was immediately measured on a SPECTRA MAX 190 plate reader at 412 nm during 5 minutes. The obtained changes (kinetic mode) were compared with daily made GSH and GSSG standards of known concentrations. All chemicals used were purchased from Sigma-Aldrich, UK or Melford, UK. The assays were further validated by measuring a subset of individuals twice. The across and within-assay repeatability was tested by using a restricted maximum likelihood (REML) mixed-model with individual identity as a random factor. The across-assay repeatability for tGSH was 78% (tGSH, n = 54, variance components ± SE, ID: 0.29 ×10^-3^ ± 0.09 ×10^-3^, residual: 0.08 ×10^-3^ ± 0.02 ˣ10^-3^), and for GSSG it was 71% (GSSG, n = 48, variance components ± SE, ID: 10.60 ×10^-6^ ± 3.79 ˣ10^-6^, residual: 4.22 ×10^-6^ ± 1.23ˣ 10^-6^). Within-assay repeatabilities were 98% and 95%, respectively. Furthermore, GSH concentration is higher in red blood cells compared to extra-cellular plasmatic fluids (Adult great tits: Mean (μM) ± SD; red blood cells: 15.36 ± 5.86, plasma: 3.56 ± 1.81). Thus, to control for the number of red blood cells in the blood, haemoglobin concentration was measured using Drabkin’s regent (0.05 g KCN, 0.2 g K_3_Fe(CN)_6_ and 1 g NaHCO_3_ for 1 liter dH_2_0). However, neither haematocrit nor haemoglobin showed a significant effect on tGSH (hematocrit; n = 303, F = 0.048, p = 0.826, haemoglobin; n = 284, F = 0.019, p = 0.890), thus was not considered any further. Laboratory day significantly influenced both tGSH and GSSG (p < 0.001), thus measures needed to be standardized (see data handling and statistics).

#### Reactive Oxygen Metabolites (ROM)

ROMs are generated by oxidation of hydroperoxides (ROOH). This oxidation occurs in the early phase of an oxidative cascade, which means that ROMs can further cause damage by generating more toxic ROS [55]. Therefore ROMs can directly related to the amount of ROS and susceptibility to ROS [55,59]. The d-ROMs test (Diacron, Grosseto, Italy) was used to detect the generated ROM in plasma. With minor modifications, the protocol provided by the manufacturer was followed. Briefly, a master mix of 1:100 of chromogen and acidic buffer was prepared, and 200 μl were added to each well on a 96-microplate. 4 μl of plasma was added and then incubated at 37°C for 75 min. Samples were analysed at 490 nm (Biotech Power, Northwest Science LTD). Each plate contained blank, calibrator, and standard plasma sample. The final d-ROM calculations were expressed as mg H_2_O_2_/dl. For d-ROM assay the across-assay repeatability was 93% (d-ROM, n = 64, variance components ± SE: ID = 3.79 ± 0.29, residual = 0.28 ± 0.05), and within assay repeatability was 99%. Recently, ROM was shown to be sensitive to time of sampling [60]. Here, however, time did not influence ROM concentration (F_1,243_ = 0.89, p = 0.35), and was therefore not controlled for.

### Data handling and statistics

#### Parameters and the rational for inclusion in models

The aim of the present study is to investigate which factors that explain variance in malaria infection in great tits and their relative importance (see below). The factors included are described in detail below. Two environmental factors were explored: *habitat quality* (evergreen *versus* deciduous-tree dominated plots) and *spring date*. Since the present study was conducted in the spring (before most vectors emerge), the majority of infections are likely to be relapses from previous infections [31]. However, later in the spring the likelihood of obtaining a primary infection increases. Thus, we expect *Plasmodium* infection to increase with spring date. For habitat quality, we predict that birds breeding in the low quality (ever-green) habitats to have a higher infection rate, since these birds are generally in poorer condition [52].

Five demographic and host intrinsic parameters were explored: *host density* - an increase in *Plasmodium* infection was predicted as an indirect effect of high host density [1,12,34]. *Sex -* previous results show mixed results. Thus, no overall directional sex effect can be predicted [1,42]. *Age –* over time the probability to be bitten by an infected vector is likely to increase [7]. In addition, parasite resistance declines with age due to immunosenescence [61]. Based on that we predicted older birds to have a higher proportion of infected individuals than the first year breeders [1]. *Body mass –* we predicted infected birds had a have lower mass (see also above); and *clutch size* - chronic malaria infection has been related to fitness costs [32], hence we predicted that prevalent birds had a smaller clutch size.

Three oxidative stress parameters were explored; *tGSH* – if the majority of infections are relapses rather than primary infections (see above), we predicted tGSH to be up-regulated in infected birds [23,26,27]. Since nothing is yet known about *P. circumflexum* and *P. relictum* and GSH the prediction is based on *P. falciparum* and GSH. During primary infection the tGSH has been shown to be down-regulated to increase cellular ROS, thereby increasing the cellular oxidative stress. However, to have a high oxidative stress under a prolonged time is likely to be more damaging than beneficial for the host due the damaging nature of ROS to anything in its surroundings, thus tGSH is predicted to be at normal levels again. Potentially, it is higher than normal to protect the cell from ROS generated from parasite metabolism [54]. In addition, *P. falciparum* is known to control the intracellular tGSH and GSSG:tGSH redox homeostasis, thus during chronic infection it is most likely the interest of both host and parasite to have a high tGSH. Hence the prediction that tGSH is up-regulated. *GSSG* – is the oxidized form of GSH, thus a high GSSG would indicate a high antioxidant activity of GSH but also high exposure to ROS, we predicted that a relapse infection is positively associated with GSH redox activity (there was a negative association between GSH (reduced form) and GSSG, r = -0.626, p < 0.0001), higher GSSG in prevalent birds compared to if uninfected is predicted. We did not include the GSH:GSSG ratio in the models due to its high correlation with spring date, tGSH and GSSG, however, as can be seen in the Additional file 3 there is no association with prevalence patterns when analyzed separately. *ROM* – if chronic malaria infection is physiologically challenging (i.e., no up-regulation of tGSH or antioxidant activity of GSH) we predicted ROM to be higher in prevalent birds [59,60]. In addition, some key biologically relevant interactions were tested such as age × antioxidant response and sex × clutch size. These are summarized in additional material (Additional files 4 and 5). All oxidative stress parameters were standardized for laboratory day to control for daily variation in chemical reaction rates (i.e., all solutions were made fresh and from scratch each day). Since a model selection approach was used (see below) to analyse the data, it is not appropriate to control for this by adding laboratory day as a covariate i.e., that would generate models that control and others that do not control for this artefact. Instead all concentrations were standardized for laboratory day by subtracting the lab day mean from the raw value and dividing that by the standard deviation of that day; this dimensionless quantity (the z-score) is commonly used in statistical analysis [62]. All samples were assayed randomly, thus it is unlikely that the standardization will bias the results.

#### Akaike information criteria (AIC) modeling

A model selection approach (AIC) was used to find the model (or models) that explain most of the variation found in infection (Tables 2 and 3). This analysis compares all possible combination of models in a given data set and ranks them by their AIC value; the model with the lowest AIC value is the best fitting model [63,64]. To account for the number of parameters included, AICc is a stricter estimate of AIC and therefore used here [63]. To evaluate the relative importance of single parameters we performed a model average calculation based on all models with a Δ value < 4 (Δ AIC = AICc specific model minus the AICc from the best fitted model [lowest AICc]; [65], but see also Additional file 3). The relative likelihood of each model was estimated by normalized Δ AICc weights (*ω*), an index of relative plausibility. It should be noted that the model selection approach is not based on direct hypothesis-testing (although, we have predictions about the directions of the effects, see above); thus parameters included in the best fitted model do not necessarily have a significant effect. For *Plasmodium* infection, species-specific models were run (Tables 2 and 3). For the top ten models including the biologically relevant interactions, the estimates and confidence intervals are provided in the additional material (Additional files 4 and 5). Nest box was fitted as random effect and the model was run with binomial distribution for infection with logit link function. Initially, habitat plot (12 areas) was also included as a random factor, but since it only explained between 0-1% of variation it was removed to simplify models. There were no differences in the final results whether body mass or body condition (residuals from the regression between tarsus and mass) was included in the models. For simplification the raw data of mass was used instead of condition. All models were sensitive to missing values, thus only individuals with all target traits were included in the AIC approximation. The reduced sample sizes are given in Tables 2 and 3. The AIC modeling was performed in R 2.12.2 [66] and the other statistical tests in JMP 8 (SAS Institute. Inc., Cary, US).

## Competing interests

The authors declare that they have no competing interests.

## Authors’ contributions

CI and BS conceived and designed the study, CI collected the data, CI and VB performed the laboratory work. CI and IS analysed the data. CI, IS and BS wrote the manuscript. All authors read and approved the final manuscript.

## Supplementary Material

Additional file 1 **Correlational analysis between all the covariates that are used in the model for*Plasmodium* prevalence.** In addition, GSH:GSSG ratio are shown here, but since it correlates with spring date and GSSG and tGSH it was not included in the models. Click here for file

Additional file 2 **Schematic picture of the experimental design of breeding density in Bagley Woods/Oxfordshire.** Each dot indicates a nest box and each habitat plot is separated with lines. Click here for file

Additional file 3 **This is the results from single parameter analysis using generalized linear models (GLM) with binomial distribution and logit link function.** All parameters, except GSH:GSSG ratio, were included in the AIC models presented in the paper (Tables 2 and 3). (DOCX 15 kb) Click here for file

Additional file 4 ***P. relictum* prevalence.** Click here for file

Additional file 5 ***P. circumflexum*prevalence.** Click here for file
