# Supplementary material for: Explaining variance of avian malaria infection in the wild: the importance of host density, habitat, individual life-history and oxidative stress
Source: BMC Ecol. 2013 Apr 8;13:15. doi: 10.1186/1472-6785-13-15 (PMC3639228; doi:10.1186/1472-6785-13-15)
Supplement: Additional file 1 — Correlational analysis between all the covariates that are used in the model forPlasmodium prevalence. In addition, GSH:GSSG ratio are shown here, but since it correlates with spring date and GSSG and tGSH it was not included in the models. [file 1472-6785-13-15-S1.docx]

Additional file 4

|  | ROM | stdGSSG | stdtGSH | Clutch size | Mass | Spring date | GSH:GSSG |
| --- | --- | --- | --- | --- | --- | --- | --- |
| ROM |  | r = -0.057, p = 0.365 | r = -0.112, p = 0.071 | r = 0.013, p = 0.844 | r = 0.079, p = 0.211 | r = 0.040, p = 0.531 | r = -0.011, p = 0.860 |
| stdGSSG | r = -0.057, p = 0.365 |  | r = -0.006, p = 0.921 | r = 0.065, p = 0.279 | r = 0.037, p = 0.523 | r = -0.101, p = 0.084 | **r = 0.380, p < 0.0001** |
| stdtGSH | r = -0.112, p = 0.071 | r = -0.006, p = 0.921 |  | r = -0.017, p = 0.771 | r = -0.064, p = 0.277 | r = -0.058, p = 0.322 | **r = 0.374, p < 0.0001** |
| Clutch size | r = 0.013, p = 0.844 | r = 0.065, p = 0.279 | r = -0.017, p = 0.771 |  | r = 0.017, p = 0.774 | r = -0.023, p = 0.698 | r = -0.013, p = 0.834 |
| Mass | r = 0.079, p = 0.211 | r = 0.037, p = 0.523 | r = -0.064, p = 0.277 | r = 0.017, p = 0.774 |  | r= 0.015, p = 0.799 | r = -0.040, p = 0.504 |
| Spring date | r = 0.040, p = 0.531 | r = -0.101, p = 0.084 | r = -0.058, p = 0.322 | r = -0.023, p = 0.698 | r= 0.015, p = 0.799 |  | **r = -0.293, p < 0.0001** |
| GSH:GSSG | r = -0.011, p = 0.860 | **r = 0.380, p < 0.0001** | **r = 0.374, p < 0.0001** | r = -0.013, p = 0.834 | r = -0.040, p = 0.504 | **r = -0.293, p < 0.0001** |  |
