# Supplementary figures and images for: Explaining variance of avian malaria infection in the wild: the importance of host density, habitat, individual life-history and oxidative stress
Source: BMC Ecol. 2013 Apr 8;13:15. doi: 10.1186/1472-6785-13-15 (PMC3639228; doi:10.1186/1472-6785-13-15)

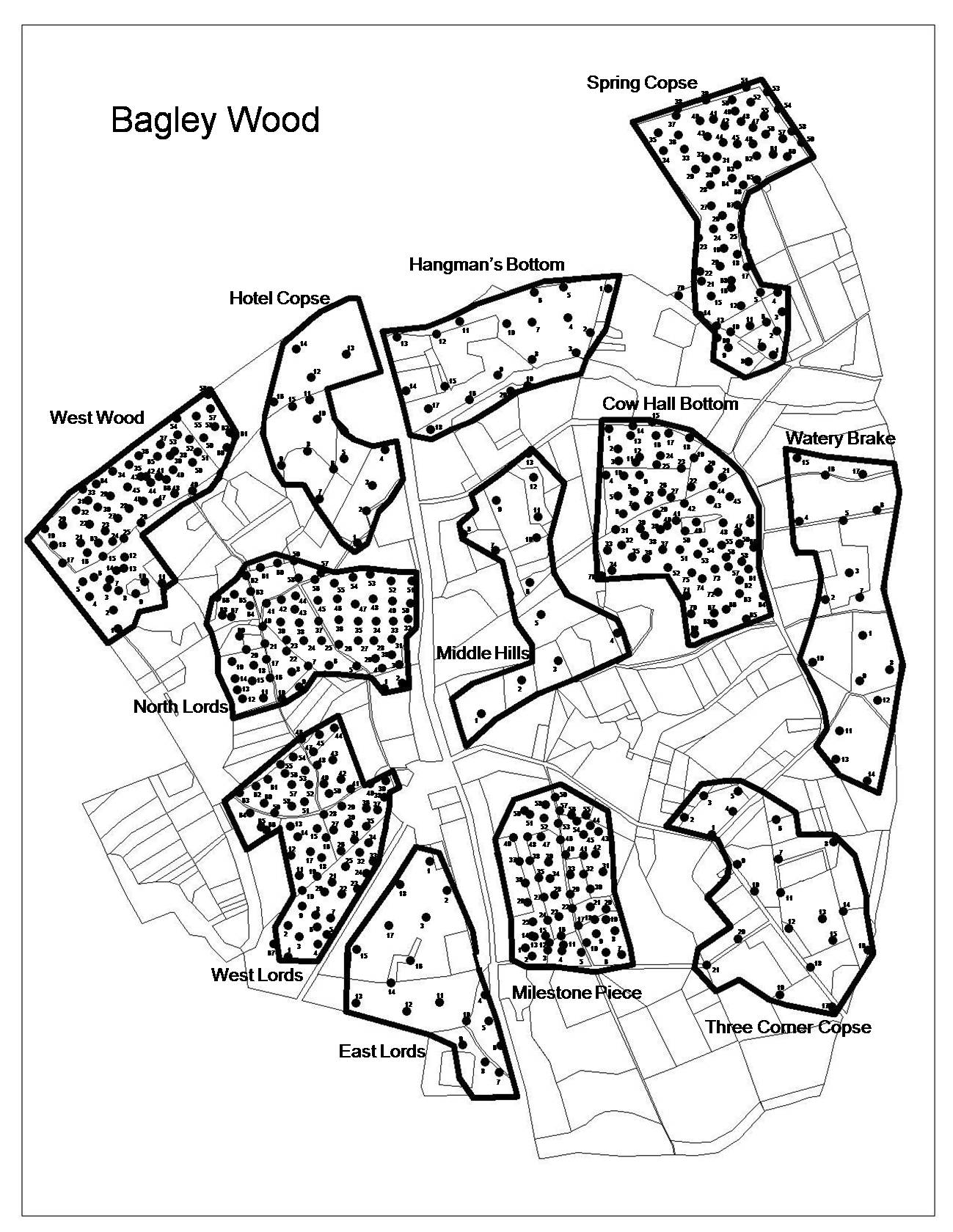

Supplement: Additional file 2 — Schematic picture of the experimental design of breeding density in Bagley Woods/Oxfordshire. Each dot indicates a nest box and each habitat plot is separated with lines. [file 1472-6785-13-15-S2.jpeg]
