# Supplementary material for: Explaining variance of avian malaria infection in the wild: the importance of host density, habitat, individual life-history and oxidative stress
Source: BMC Ecol. 2013 Apr 8;13:15. doi: 10.1186/1472-6785-13-15 (PMC3639228; doi:10.1186/1472-6785-13-15)
Supplement: Additional file 3 — This is the results from single parameter analysis using generalized linear models (GLM) with binomial distribution and logit link function. All parameters, except GSH:GSSG ratio, were included in the AIC models presented in the paper (Tables 2 and 3). (DOCX 15 kb) [file 1472-6785-13-15-S3.docx]

Additional file 5

|  | *P.circumflexum* | | | *P.relictum* | | |
| --- | --- | --- | --- | --- | --- | --- |
|  | **Parameter esti. ± Std Err** | **Chi-square** | **P-value** | **Parameter esti. ± Std Err** | **Chi-square** | **P-value** |
| age | 0.108 ± 0.164 | 0.430 | 0.512 | 0.252 ± 0.147 | 3.027 | 0.082 |
| sex | -0.066 ± 0.164 | 0.163 | 0.687 | 0.253 ± 0.145 | 3.114 | 0.078 |
| mass | -0.013 ± 0.182 | 0.005 | 0.943 | -0.112 ± 0.156 | 0.518 | 0.472 |
| spring date | -0.004 ± 0.045 | 0.009 | 0.924 | -0.074 ± 0.040 | 3.538 | 0.060 |
| clutch size | -0.096 ± 0.110 | 0.776 | 0.379 | -0.200 ± 0.096 | 4.456 | 0.035 |
| Q | 0.038 ± 0.184 | 0.042 | 0.837 | -0.101 ± 0.165 | 0.380 | 0.538 |
| D | -0.419 ± 0.197 | 5.043 | 0.025 | 0.017 ± 0.147 | 0.013 | 0.911 |
| stdtGSH | -0.046 ± 0.168 | 0.022 | 0.882 | 0.111 ± 0.142 | 2.490 | 0.115 |
| stdGSSG | -0.335 ± 0.183 | 3.396 | 0.065 | 0.087 ± 0.145 | 0.448 | 0.503 |
| ROM | -0.030 ± 0.084 | 0.120 | 0.729 | -0.153 ± 0.113 | 1.165 | 0.199 |
| GSH/GSSG | -0.030 ± 0.055 | 1.004 | 0.316 | 0.013 ± 0.043 | 0.046 | 0.830 |
