# Supplementary material for: Explaining variance of avian malaria infection in the wild: the importance of host density, habitat, individual life-history and oxidative stress
Source: BMC Ecol. 2013 Apr 8;13:15. doi: 10.1186/1472-6785-13-15 (PMC3639228; doi:10.1186/1472-6785-13-15)
Supplement: Additional file 4 — P. relictum prevalence. [file 1472-6785-13-15-S4.doc]

Additional file 2.

| ***P.relictum* prevalence** | |  |  |  |  |
| --- | --- | --- | --- | --- | --- |
|  | Parameters | Deviance | AICc | ∆AICc | *ω* |
| Model 1 | 1+2+9+10+13+17 | 260.2 | 276.8 | 0 | 0.01 |
| Model 2 | 1+2+9+10+13 | 262.45 | 276.91 | 0.12 | 0.01 |
| Model 3 | 1+2+9+10+13+14+17 | 258.61 | 277.35 | 0.56 | 0.01 |
| Model 4 | 1+2+4+9+10+13 | 260.86 | 277.45 | 0.65 | 0.01 |
| Model 5 | 1+2+4+9+10+13+17 | 258.72 | 277.46 | 0.66 | 0.01 |
| Model 6 | 1+2+9 | 267.23 | 277.47 | 0.67 | 0.01 |
| Model 7 | 1+2+8+9+10+13 | 260.95 | 277.54 | 0.75 | 0.01 |
| Model 8 | 1+2+9+10+13+14 | 260.97 | 277.56 | 0.76 | 0.01 |
| Model 9 | 1+2+8+9+10+13+17 | 258.86 | 277.6 | 0.81 | 0.01 |
| Model 10 | 1+2+4+8+9 | 263.2 | 277.66 | 0.86 | 0.01 |
| All variables included: 1 = age, 2 = clutch size(cs), 3 = density (D), 4 = date, 5 = GSSG, 6 = mass, 7 = habitat quality (Q), 8 = ROM, 9 = sex, 10 = tGSH, 11 = age×cs, 12 = age×D, 13 = age×tGSH, 14 = sex×cs, 15 = D×sex, 16 = D×tGSH, 17 = sex×tGSH, 18 = age×ROM, 19 = age×sex, 20 = D×ROM, 21 = ROM×sex | | | | | |
| **Parameters** | **Estimate** | **SE** | **Z** | **Pr(>|z|)** |  |
| **Model 1** |  |  |  |  |  |
| (Intercept) | -1.160 | 1.139 | -1.018 | 0.308 |  |
| age(y) | 0.330 | 0.357 | 0.925 | 0.355 |  |
| sex(m) | 0.049 | 0.360 | 0.136 | 0.892 |  |
| tGSH | -0.007 | 0.343 | -0.021 | 0.983 |  |
| Cs | -0.081 | 0.119 | -0.680 | 0.497 |  |
| age(y)×tGSH | -0.079 | 0.372 | -0.212 | 0.832 |  |
| sex(m)×tGSH | -0.190 | 0.377 | -0.504 | 0.615 |  |
| Box (random): Variance = 4.784e-09, Std.dev = 6.917e-05  y = young, m = male | | | |  |  |
| **Model 6 (simplest)** | | | | | |
| (Intercept) | -1.148 | 1.122 | -1.023 | 0.306 |  |
| age(y) | 0.307 | 0.353 | 0.870 | 0384 |  |
| sex(m) | 0.083 | 0.352 | 0.234 | 0.815 |  |
| Cs | -0.081 | 0.117 | -0.688 | 0.491 |  |
| Box (random): Variance = 8.742e-11, Std.dev = 9.350e-06 | | | |  |  |
