# Supplementary material for: Explaining variance of avian malaria infection in the wild: the importance of host density, habitat, individual life-history and oxidative stress
Source: BMC Ecol. 2013 Apr 8;13:15. doi: 10.1186/1472-6785-13-15 (PMC3639228; doi:10.1186/1472-6785-13-15)
Supplement: Additional file 5 — P. circumflexumprevalence. [file 1472-6785-13-15-S5.docx]

Additional file 3.

| ***P.circumflexum* prevalence** | |  |  |  |  |
| --- | --- | --- | --- | --- | --- |
|  | Parameters | Deviance | AICc | ∆AICc | *Ω* |
| Model 1 | 1+2+4+9 | 210.39 | 222.72 | 0 | 0.06 |
| Model 2 | 1+2+4+8+9+15 | 206.60 | 223.17 | 0.44 | 0.05 |
| Model 3 | 1+2+9 | 213.73 | 223.96 | 1.24 | 0.03 |
| Model 4 | 1+2+8+9+15 | 209.66 | 224.1 | 1.38 | 0.03 |
| Model 5 | 1+2+4+7+9 | 210.01 | 224.46 | 1.73 | 0.03 |
| Model 6 | 1+2+4+6+9 | 210.06 | 224.51 | 1.78 | 0.03 |
| Model 7 | 1+2+4+8+9 | 210.09 | 224.54 | 1.81 | 0.03 |
| Model 8 | 1+2+3+4+9 | 210.27 | 224.71 | 1.99 | 0.02 |
| Model 9 | 1+2+4+5+9 | 210.28 | 224.72 | 2 | 0.02 |
| Model 10 | 1+2+4+7+9+14 | 208.32 | 224.9 | 2.17 | 0.02 |
| All variables included: 1 = age, 2 = density (D), 3 = date, 4 = GSSG, 5 = habitat quality (Q), 6 = ROM, 7 = sex, 8 = tGSH, 9 = age×D, 10 = age×ROM, 11 = age×sex, 12 = age×tGSH, 13 = D×ROM, 14 = D×sex, 15 = D×tGSH, 16 = ROM×sex, 17 = sex×tGSH, 18 = age×cs, 19 = sex×cs, 20 = cs, 21 = mass | | | | | |
| **Parameters** | **Estimate** | **SE** | **Z** | **Pr(>\|z\|)** |  |
| **Model 1** |  |  |  |  |  |
| (Intercept) | -1.406 | 0.262 | -5.367 | 0.000 |  |
| age(y) | -0.152 | 0.395 | -0.384 | 0.701 |  |
| D(low) | -2.571 | 1.044 | -2.462 | 0.014 |  |
| GSSG | -0.353 | 0.199 | -1.770 | 0.077 |  |
| age(y)×D(low) | 2.582 | 1.166 | 2.215 | 0.027 |  |
| Box (random): Variance = 0.000, Std.dev = 0.000 | | | |  |  |
| **Model 3 (simplest)** | | | | | |
| (Intercept) | -1.360 | 0.257 | -5.287 | 0.000 |  |
| age(y) | -0.129 | 0.392 | -0.328 | 0.743 |  |
| D(low) | -2.552 | 1.042 | -2.449 | 0.014 |  |
| age(y)×D(low) | 2.490 | 1.161 | 2.145 | 0.032 |  |
| Box (random): Variance = 0.000, Std.dev = 0.000 | | | |  |  |
